# Supplementary material for: Environmental and societal costs of maize production decrease by addressing the uncertainty in nitrogen rate recommendations
Source: Nat Commun. 2026 Feb 5;17:2375. doi: 10.1038/s41467-026-68988-y (PMC12982502; doi:10.1038/s41467-026-68988-y)
Supplement: Supplementary file 1 — Supplementary Information [file 41467_2026_68988_MOESM1_ESM.pdf]

Supplementary Information

Environmental and societal costs of maize production decrease by addressing the uncertainty in nitrogen rate recommendations

Francisco Palmero[1]*, Eric A. Davidson[2], Kaiyu Guan[3,4], Alison J. Eagle[5], Hannah E. Birgé[6], P.V. Vara Prasad[7], Trevor J. Hefley[8], Jeffrey R. Schussler[9], Ignacio A. Ciampitti[1]*

[1] Department of Agronomy, Purdue University, West Lafayette, Indiana, 47907, USA

[2] Appalachian Laboratory, University of Maryland Center for Environmental Science, Frostburg, Maryland, 2153, USA.

[3] National Center for Supercomputing Center, University of Illinois at Urbana Champaign, Urbana, Illinois, 61801, USA.

[4] Department of Natural Resources and Environmental Sciences, University of Illinois at Urbana Champaign, Urbana, Illinois, 61801, USA.

[5] Environmental Defense Fund, Raleigh, North Carolina, 27607, USA.

[6] The Nature Conservancy, Arlington, Virginia, 22203, USA.

[7] Department of Agronomy, Kansas State University, Manhattan, Kansas, 66506, USA.

[8] Department of Statistics, Kansas State University, Manhattan, Kansas, 66506, USA.

[9] Schussler Ag Research Solutions, Marion, Iowa, 52302, USA.

*Corresponding authors.

Email addresses: fpalmero@purdue.edu (F. Palmero), iciampit@purdue.edu (I.A. Ciampitti)

Supplementary Information

1. **Supplementary Tables**
2. **Supplementary Figures**
3. **References**

1. Supplementary Tables

Table S1. Prior probability distributions of the parameters in the quadratic plateau models fitted to the relationship between grain yield and nitrogen rates. Inside the parenthesis for the hyperparameters column, the left and the right numbers indicate the shape and the rate of the distribution, respectively.

State	Parameter	Probability Distribution	Hyperparameters
Iowa	β_0	gamma	(10.89, 0.0015)
	β_1		(3.26, 0.0467)
	β_2		(1.5, 10.00)
	σ_ε		(3.75, 0.0025)
Illinois	β_0	gamma	(9.389, 0.0014)
	β_1		(2.82, 0.043)
	β_2		(0.96, 8.00)
	σ_ε		(3.75, 0.0025)
Indiana	β_0	gamma	(10.43, 0.0015)
	β_1		(2.64, 0.039)
	β_2		(1.22, 8.75)
	σ_ε		(3.75, 0.0025)
Minnesota	β_0	gamma	(9.041, 0.0015)
	β_1		(2.16, 0.045)
	β_2		(1.025, 11.39)
	σ_ε		(6.51, 0.0042)
Missouri	β_0	gamma	(13.68, 0.0021)
	β_1		(1.98, 0.0415)
	β_2		(1.20, 7.083)
	σ_ε		(8.026, 0.0044)

Table S1 (continued).

State	Parameter	Probability Distribution	Hyperparameters
North Dakota	β_0	gamma	(13.26, 0.0018)
	β_1		(0.914, 0.0307)
	β_2		(0.916, 8.33)
	σ_ε		(9.17, 0.0042)
Nebraska	β_0	gamma	(20.28, 0.00205)
	β_1		(1.838, 0.0416)
	β_2		(0.89, 8.14)
	σ_ε		(8.105, 0.0043)
Wisconsin	β_0	gamma	(20.17, 0.00217)
	β_1		(1.703, 0.0382)
	β_2		(1.071, 7.1428)
	σ_ε		(5.266, 0.00315)

Table S2. Reason justifying why the sites in this table were not included to analyze nitrogen reductions. AONR stands for the agronomic optimum nitrogen rate, E[AONR] is the expected value of the AONR. N rate represents the nitrogen fertilization rate applied to maize crop at planting. EONR represents the economic optimum nitrogen rate. $\hat{R}$ ("R hat") indicates the Gelman-Rubin diagnostic. The column site indicates the number of the experiment in the original dataset.

State	Site	Reason
Illinois	T3	E[AONR] > max(N rate)
Minnesota	T39	E[AONR] > max(N rate)
Missouri	T25	E[AONR] > max(N rate) and $\hat{R}$ >1.02 for EONR
Missouri	T26	E[AONR] > max(N rate) and $\hat{R}$ >1.02 for EONR
North Dakota	T12	E[AONR] > max(N rate) and $\hat{R}$ >1.02 for EONR
North Dakota	T27	$\hat{R}$ >1.02 for EONR
North Dakota	T28	$\hat{R}$ >1.02 for EONR
North Dakota	T44	$\hat{R}$ >1.02 for AONR and EONR
North Dakota	T45	$\hat{R}$ >1.02 for AONR and EONR
Nebraska	T13	E[AONR] > max(N rate) and $\hat{R}$ >1.02 for EONR
Nebraska	T29	E[AONR] > max(N rate) and $\hat{R}$ >1.02 for EONR
Nebraska	T30	$\hat{R}$ >1.02 for AONR and EONR
Nebraska	T47	$\hat{R}$ >1.02 for AONR and EONR
Wisconsin	T31	$\hat{R}$ >1.02 for EONR
Wisconsin	T32	E[AONR] > max(N rate) and $\hat{R}$ >1.02 for EONR
Wisconsin	T48	$\hat{R}$ >1.02 for EONR
Wisconsin	T49	$\hat{R}$ >1.02 for AONR and EONR

Table S3. Descriptive statistics for the retrieved studies addressing N_2O–N emissions and $NO_3^- - N$ leaching in the United States Corn Belt. The last column indicates the minimum (Min), 0.25 quantile (Q0.25), the mean, the variance, 0.75 quantile (Q0.75), and the maximum value of N_2O–N emissions and $NO_3^- - N$ leaching across all the studies.

<table>
<tr><th rowspan="2">Reference</th><th rowspan="2">Number of Obs.</th><th rowspan="2">State</th><th colspan="2">N_2O – N (kg ha^{-1})</th><th colspan="6">N_2O – N (kg ha^{-1})</th></tr>
<tr><th>Mean</th><th>Variance</th><th>Min</th><th>Q0.25</th><th>Mean</th><th>Variance</th><th>Q0.75</th><th>Max</th></tr>
<tr><td>Adviento-Borbe et al. (2007)[1]</td><td>10</td><td>NE</td><td>3.71</td><td>5.71</td><td rowspan="14">0.28</td><td rowspan="14">1.29</td><td rowspan="14">3.93</td><td rowspan="14">11.65</td><td rowspan="14">5.37</td><td rowspan="14">16.26</td></tr>
<tr><td>Fujinuma et al. (2011)[2]</td><td>6</td><td>MN</td><td>0.90</td><td>0.24</td></tr>
<tr><td>Maharjan and Venterea (2013)[3]</td><td>3</td><td>MN</td><td>2.43</td><td>0.82</td></tr>
<tr><td>Maharjan et al. (2014)[4]</td><td>3</td><td>MN</td><td>0.34</td><td>0.01</td></tr>
<tr><td>Parkin and Hatfield (2010)[5]</td><td>2</td><td>IA</td><td>6.14</td><td>1.57</td></tr>
<tr><td>Phillips et al. (2009)[6]</td><td>2</td><td>ND</td><td>0.48</td><td>0.01</td></tr>
<tr><td>Smith et al. (2011)[7]</td><td>8</td><td>IN</td><td>2.82</td><td>0.22</td></tr>
<tr><td>Venterea et al. (2010)[8]</td><td>12</td><td>MN</td><td>1.59</td><td>0.65</td></tr>
<tr><td>Hernandez-Ramirez et al. (2009)[9]</td><td>4</td><td>IN</td><td>5.65</td><td>1.08</td></tr>
<tr><td>Johnson et al. (2010)[10]</td><td>9</td><td>MN</td><td>5.29</td><td>0.77</td></tr>
<tr><td>Mitchell et al. (2013)[11]</td><td>4</td><td>IA</td><td>4.49</td><td>1.00</td></tr>
<tr><td>Omonode and Vyn (2013)[12]</td><td>8</td><td>IN</td><td>4.09</td><td>29.47</td></tr>
<tr><td>Johnson II et al. (2024)[13]</td><td>2</td><td>IA</td><td>1.01</td><td>0.12</td></tr>
<tr><td>Preza-Fontes et al. (2023)[14]</td><td>9</td><td>IL</td><td>10.40</td><td>3.29</td></tr>
</table>

Table S3 (continued).

Reference	Number of Obs.	State	$NO_3^- - N$ (kg ha^{-1})		$NO_3^- - N$ (kg ha^{-1})					
			Mean	Variance	Min	Q0.25	Mean	Variance	Q0.75	Max
Bakhsh et al. (2007)[15]	5	IA	10.1	48						
Bakhsh et al. (2010)[16]	10	IA	11.4	55						
Helmers et al. (2012)[17]	28	IA	46.7	403						
Jaynes (2013)[18]	6	IA	32.3	127						
Jaynes et al. (2001)[19]	6	IA	47.5	107						
Kucharik and Brye (2003)[20]	10	WI	56.1	3161						
Maharjan et al. (2014)[4]	3	MN	25.8	19						
Prunty and Greenland (1997)[21]	4	ND	47.2	2822						
Randall et al. (2003)[22]	24	MN	36.0	1209	0.02	14.0	34.8	1005	47.7	201.1
Randall and Vetsch (2005)[23]	24	MN	17.4	256						
Sexton et al. (1996)[24]	12	MN	60.0	1831						
Walters and Malzer (1990)[25]	24	MN	49.2	1726						
Kalita et al. (2006)[26]	21	IL	24.8	330						
O'Brien et al. (2022)[27]	10	IA	14.8	149						
Preza-Fontes et al. (2023)[14]	3	IL	21.9	24						
Johnson II et al. (2024)[13]	2	IA	26.1	283						
Gentry et al. (2023)[28]	6	IL	27.8	49						

2. Supplementary Figures

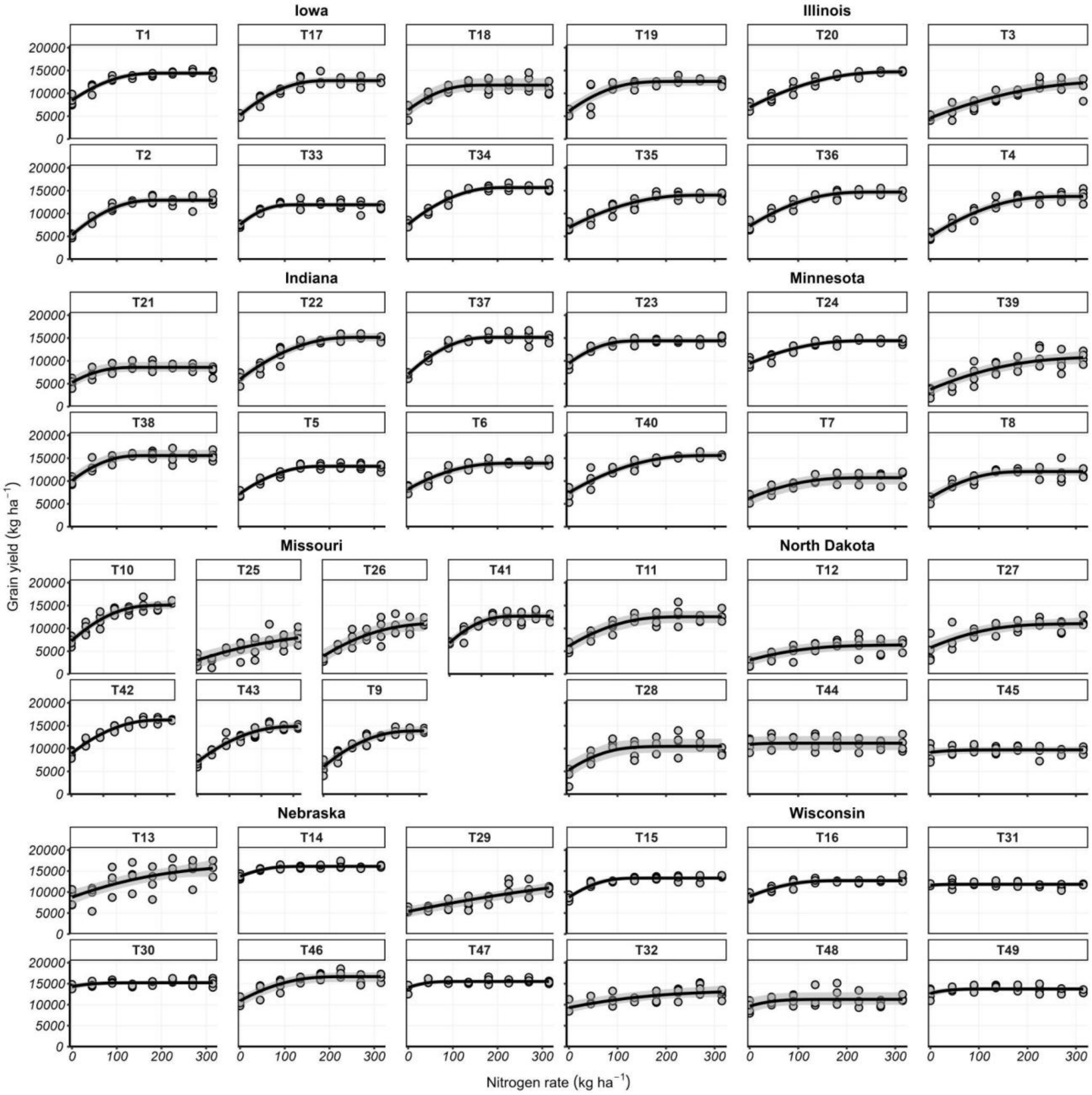

Fig. S1 | Relationship between maize grain yield (*y*) and nitrogen rate (*x*) in each of the studied sites. The solid lines represent the expected grain yield at different nitrogen rates. The shadow areas indicate the 95% credible interval of the posterior predictive distributions. The label in each plot indicates the number of the trial in the original dataset. In each site, the total number of observations was 32 (n=32) with 4 repetitions per treatment.

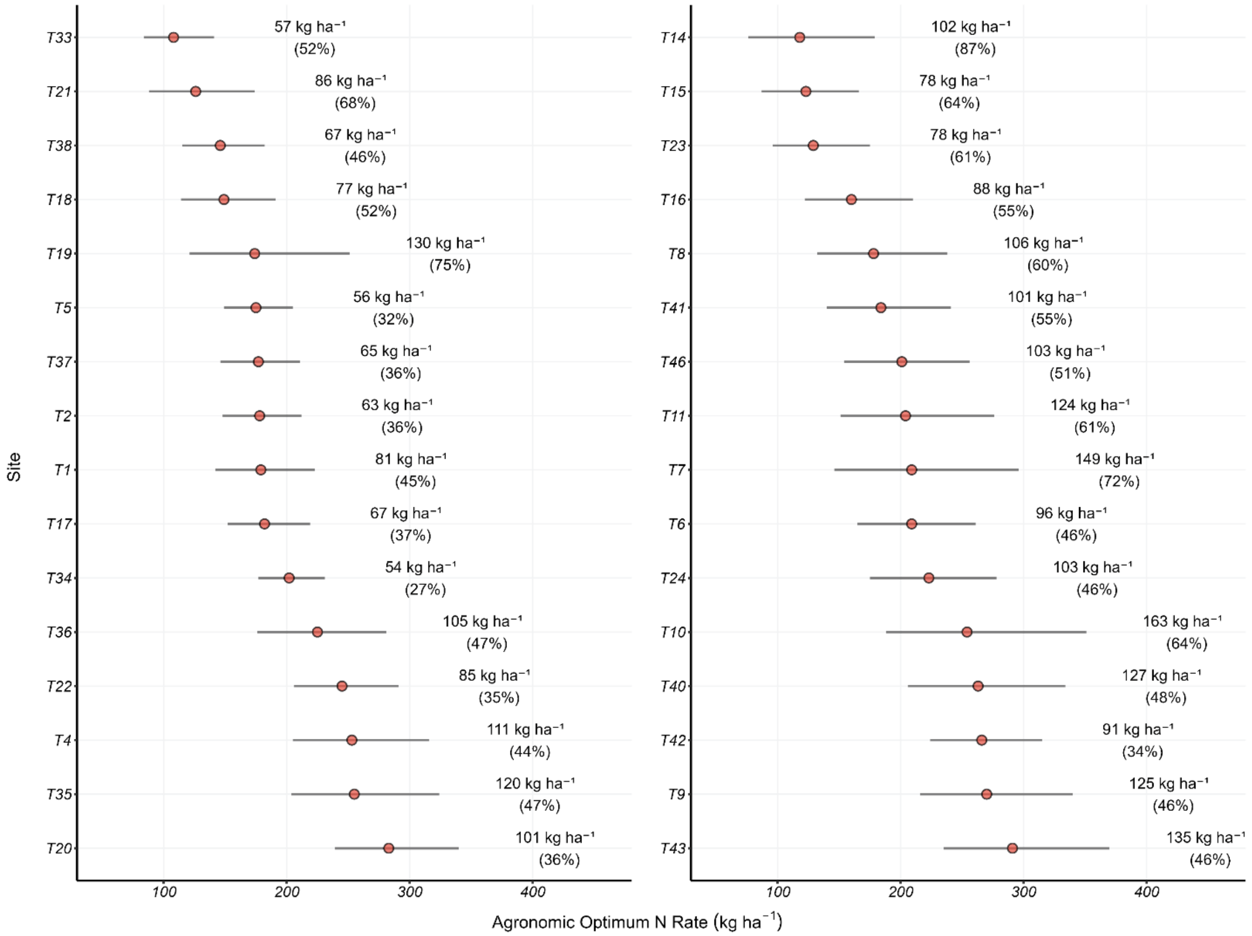

Fig. S2 | Summary of the agronomic optimum nitrogen rate (AONR) for each of the selected sites. Circles indicate the expected value of the distribution (E[AONR]), the horizontal bars and upper values on the right indicate the 95% credible interval of the AONR, and the lower values, between parentheses, on the right indicate the proportion of the uncertainty with respect to E[AONR]. The labels in the *y* axis indicate the number of the trial in the original dataset. In each site, the total number of observations was 32 (n=32) with 4 repetitions per treatment. Source data are provided as a Source Data file.

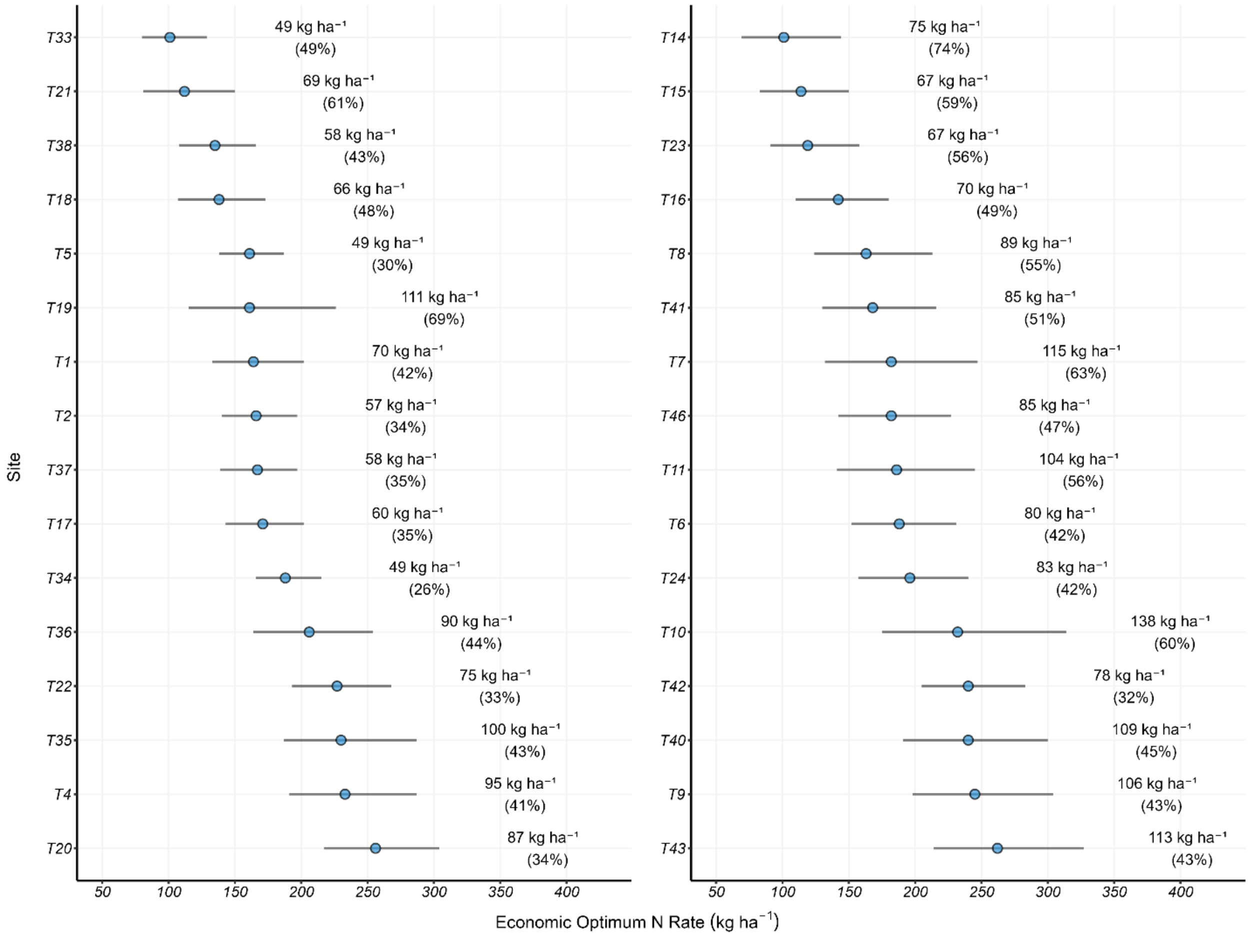

Fig. S3 | Summary of the economic optimum nitrogen rate (EONR) for each of the selected sites. Circles indicate the expected value of the distribution (E[EONR]), the horizontal bars and upper-level values on the right indicate the 95% credible interval of the EONR, and the lower-level values, between parentheses, on the right indicate the proportion of the uncertainty with respect to E[EONR]. The labels in the *y* axis indicate the number of the trial in the original dataset. In each site, the total number of observations was 32 (n=32) with 4 repetitions per treatment. Source data are provided as a Source Data file.

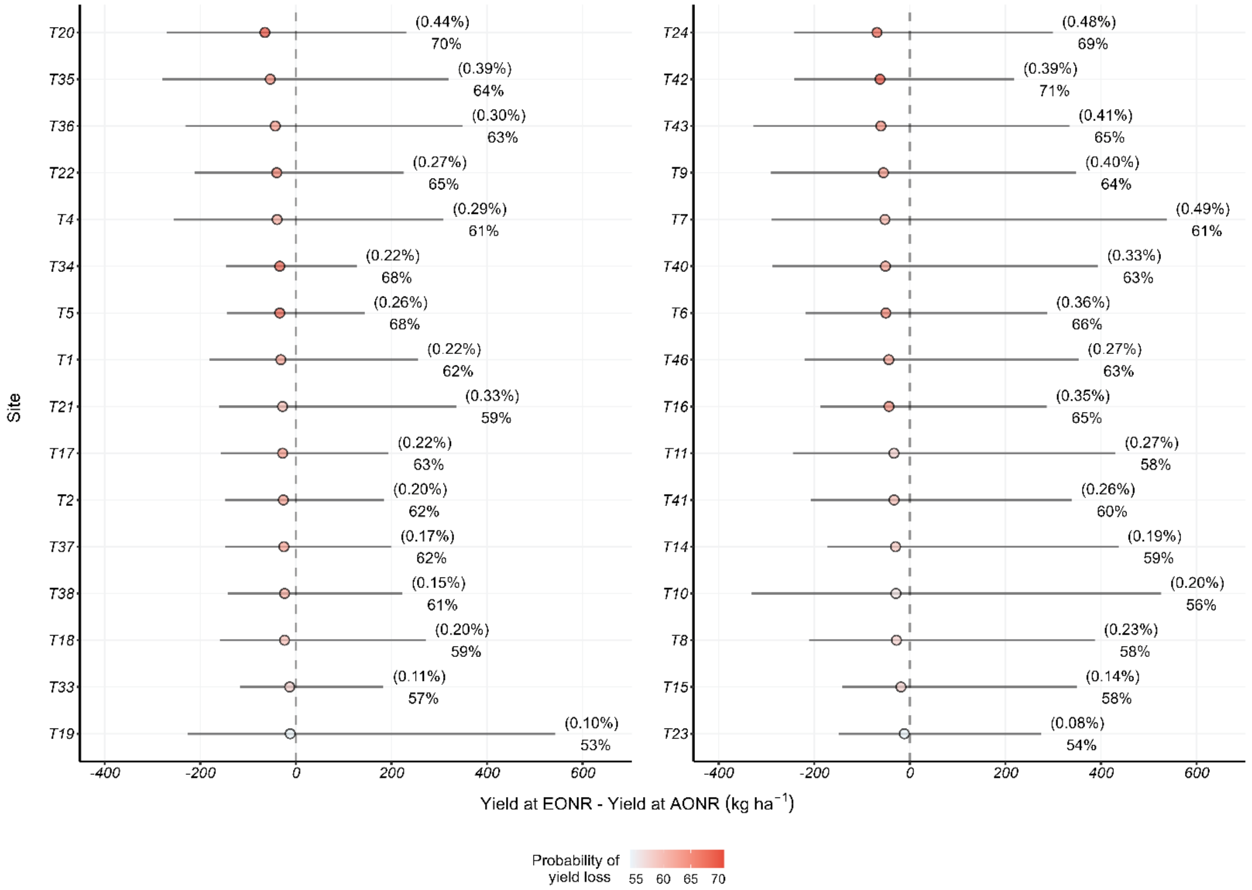

Fig. S4 | Yield loss and its probability when reducing the nitrogen rate from the expected value of the agronomic optimum nitrogen rate probability distribution (E[AONR]) to expected value of the economic optimum nitrogen rate probability distribution (E[EONR]). This corresponds to Phase I of nitrogen reductions. Circles indicate the expected yield loss, the horizontal bars and indicate the 95% credible interval of the yield loss estimation. The upper-level values, between parentheses, on the right indicate the proportion of yield loss with respect to E[AONR] and the lower-level values on the right and circle colors indicate the probability associated to each yield loss estimation. The labels in the *y* axis indicate the number of the trial in the original dataset. In each site, the total number of observations was 32 (n=32) with 4 repetitions per treatment. Source data are provided as a Source Data file.

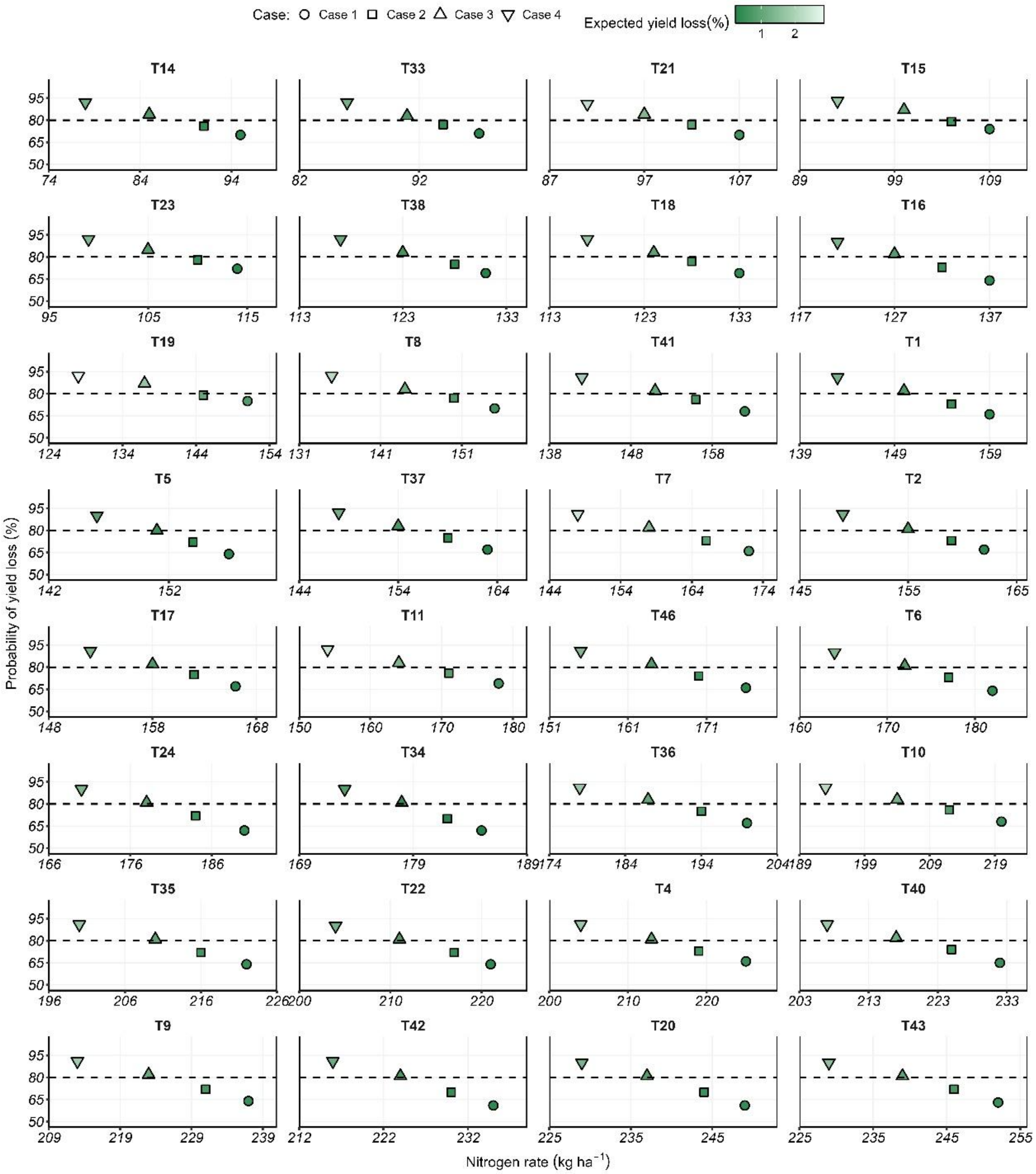

Fig. S5 | Probability of yield loss and expected yield loss with respect to yield at the economic optimum nitrogen rate (EONR) (Phase II) in a given quadratic plateau model within each site for each case of nitrogen fertilization reduction. Cases 1, 2, 3, and 4 represent the 0.4, 0.3, 0.2, and 0.1 quantiles of the EONR probability distribution, respectively. The label in each plot indicates the number of the trial in the original dataset. Source data are provided as a Source Data file.

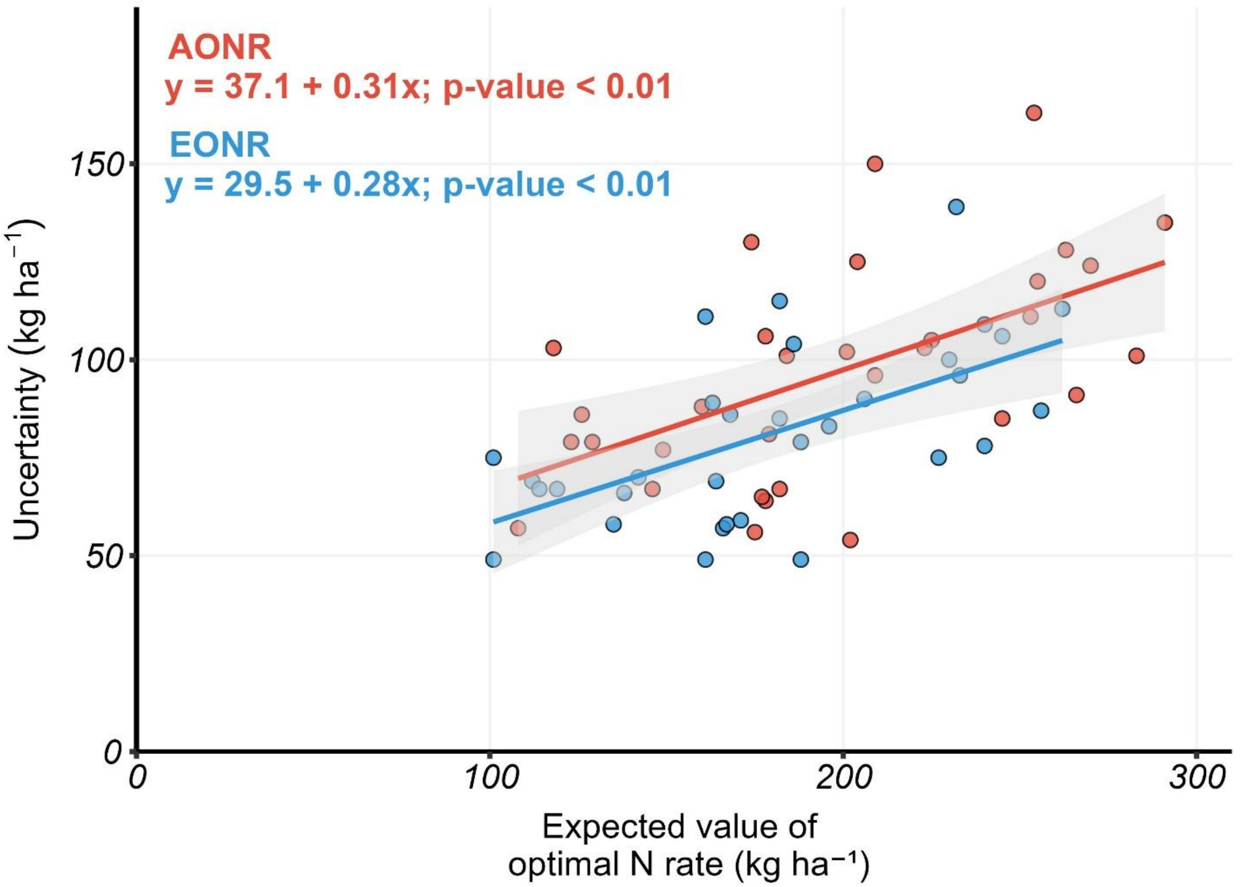

Fig. S6 | Relationship between the expected value of the optimal nitrogen rates (AONR and EONR) and their associated uncertainties. Solid line is the least square estimation of the regression between the dependent (*y*) and independent (*x*) variables in this plot. Shadow area represents the 95% confidence interval of the regression line. P-values < 0.01 indicate that the slopes were different from zero for $\alpha = 0.01$. The values of each dot were obtained with a total number of observations of 32 (n=32) with 4 repetitions per treatment. Source data are provided as a Source Data file.

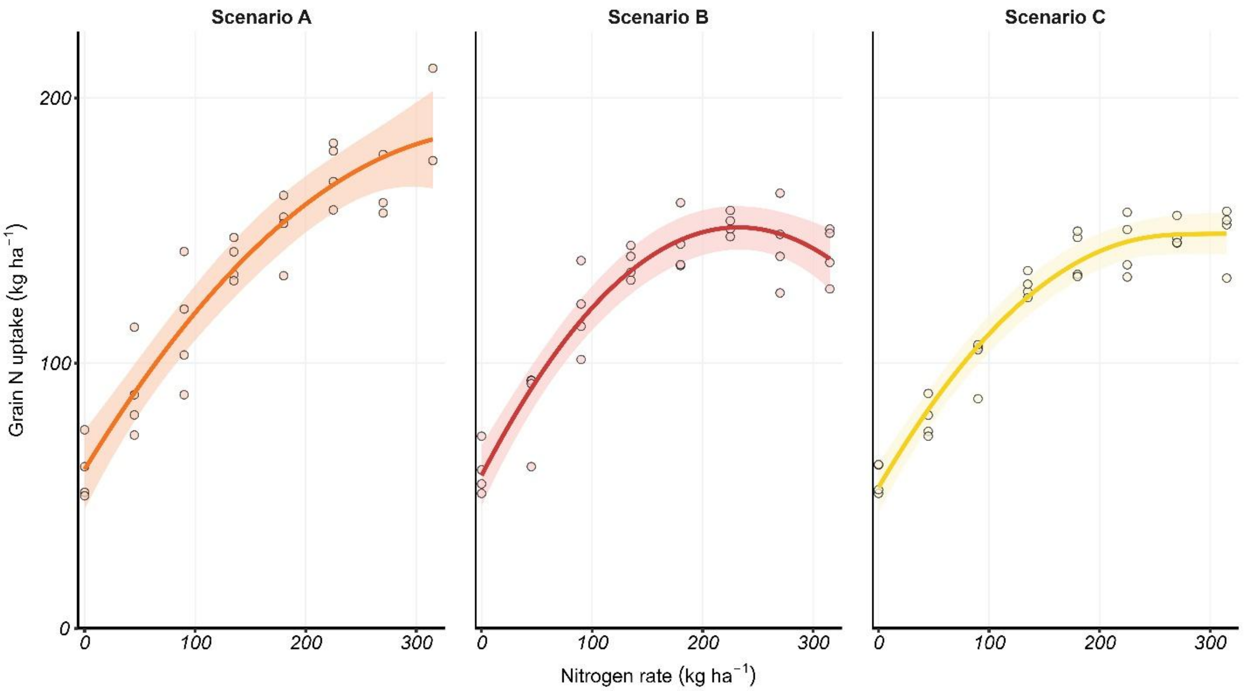

Fig. S7 | Relationship between grain nitrogen uptake (*y*) and nitrogen rate (*x*) in each of the selected scenarios. The solid lines represent the expected nitrogen uptake at different nitrogen rates. The shadow areas indicate the 95% credible interval of the posterior predictive distributions. In each scenario, the total number of observations was 32 (n=32) with 4 repetitions per treatment.

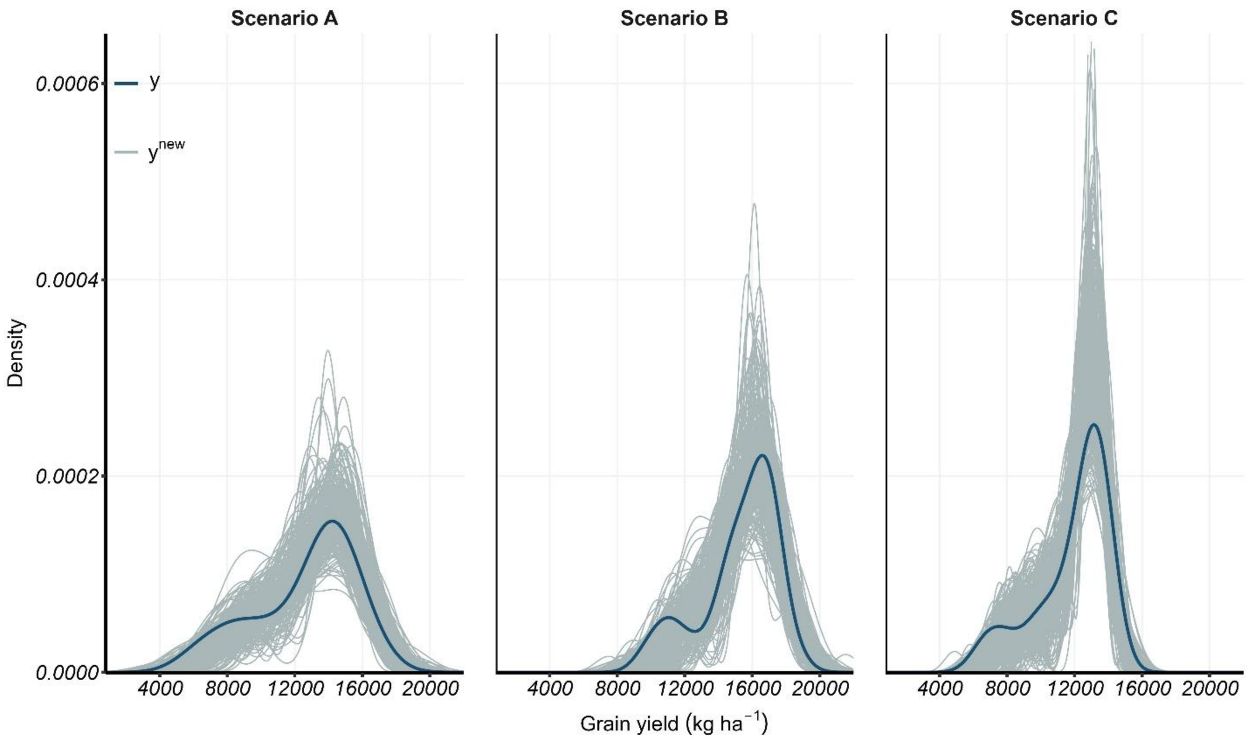

Fig. S8 | Posterior predictive distributions for the models fitted to the maize grain yield versus nitrogen rate relationship in each of the selected scenarios. The blue lines are the distribution of the observed values of grain yield. The gray lines are two hundred curves representing the distribution of 32 observations (total number of observations in each scenario) randomly sampled from the posterior predictive distributions for grain yield.

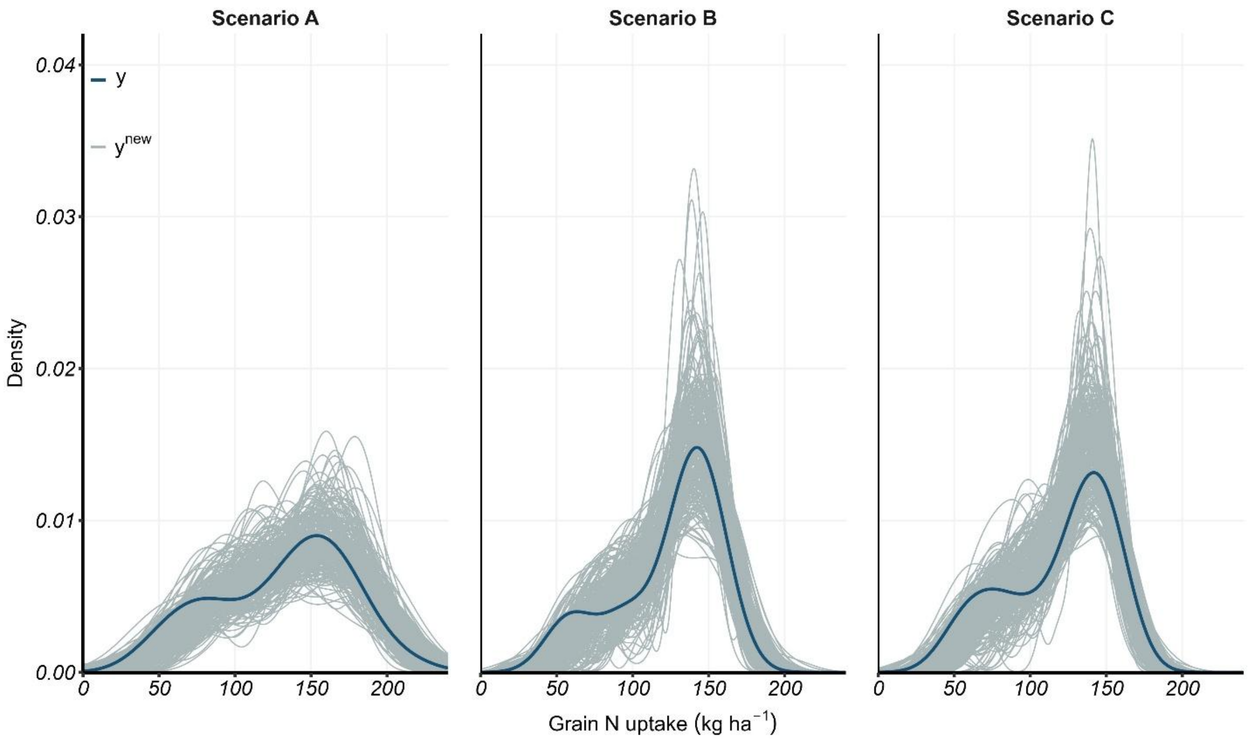

Fig. S9 | Posterior predictive distributions for the models fitted to the grain nitrogen uptake versus nitrogen rate relationship in each of the selected scenarios. The blue lines are the distribution of the observed values of grain nitrogen uptake. The gray lines are two hundred curves representing the distribution of 32 observations (total number of observations in each scenario) randomly sampled from the posterior predictive distributions for grain nitrogen uptake.

3. References

References Table S3

1. Adviento-Borbe, M. a. A., Haddix, M. L., Binder, D. L., Walters, D. T. & Dobermann, A. Soil greenhouse gas fluxes and global warming potential in four high-yielding maize systems. *Global Change Biology* **13**, 1972–1988 (2007).
2. Fujinuma, R., Venterea, R. T. & Rosen, C. Broadcast Urea Reduces N2O but Increases NO Emissions Compared with Conventional and Shallow-Applied Anhydrous Ammonia in a Coarse-Textured Soil. *Journal of Environmental Quality* **40**, 1806–1815 (2011).
3. Maharjan, B. & Venterea, R. T. Nitrite intensity explains N management effects on N2O emissions in maize. *Soil Biology and Biochemistry* **66**, 229–238 (2013).
4. Maharjan, B., Venterea, R. T. & Rosen, C. Fertilizer and Irrigation Management Effects on Nitrous Oxide Emissions and Nitrate Leaching. *Agronomy Journal* **106**, 703–714 (2014).
5. Parkin, T. B. & Hatfield, J. L. Influence of nitrapyrin on N2O losses from soil receiving fall-applied anhydrous ammonia. *Agriculture, Ecosystems & Environment* **136**, 81–86 (2010).
6. Phillips, R. L., Tanaka, D. L., Archer, D. W. & Hanson, J. D. Fertilizer Application Timing Influences Greenhouse Gas Fluxes Over a Growing Season. *Journal of Environmental Quality* **38**, 1569–1579 (2009).
7. Smith, D. R., Hernandez-Ramirez, G., Armstrong, S. D., Bucholtz, D. L. & Stott, D. E. Fertilizer and Tillage Management Impacts on Non-Carbon-Dioxide Greenhouse Gas Emissions. *Soil Science Society of America Journal* **75**, 1070–1082 (2011).
8. Venterea, R. T., Dolan, M. S. & Ochsner, T. E. Urea Decreases Nitrous Oxide Emissions Compared with Anhydrous Ammonia in a Minnesota Corn Cropping System. *Soil Science Society of America Journal* **74**, 407–418 (2010).
9. Hernandez-Ramirez, G., Brouder, S. M., Smith, D. R. & Van Scoyoc, G. E. Greenhouse Gas Fluxes in an Eastern Corn Belt Soil: Weather, Nitrogen Source, and Rotation. *Journal of Environmental Quality* **38**, 841–854 (2009).
10. Johnson, J. M. F., Archer, D. & Barbour, N. Greenhouse Gas Emission from Contrasting Management Scenarios in the Northern Corn Belt. *Soil Science Society of America Journal* **74**, 396–406 (2010).
11. Mitchell, D. C., Castellano, M. J., Sawyer, J. E. & Pantoja, J. Cover Crop Effects on Nitrous Oxide Emissions: Role of Mineralizable Carbon. *Soil Science Society of America Journal* **77**, 1765–1773 (2013).

12. Omonode, R. A. & Vyn, T. J. Nitrification Kinetics and Nitrous Oxide Emissions when Nitrapyrin is Coapplied with Urea–Ammonium Nitrate. *Agronomy Journal* **105**, 1475–1486 (2013).
13. Johnson, F. E., Roth, R. T., Ruffatti, M. D. & Armstrong, S. D. Cover crop impacts on nitrogen losses and environmental damage cost. *Agriculture, Ecosystems & Environment* **363**, 108859 (2024).
14. Preza-Fontes, G., Christianson, L. E., Greer, K., Bhattarai, R. & Pittelkow, C. M. In-season split nitrogen application and cover cropping effects on nitrous oxide emissions in rainfed maize. *Agriculture, Ecosystems & Environment* **326**, 107813 (2022).
15. Bakhsh, A., Kanwar, R. S., Pederson, C. & Bailey, T. B. N-Source Effects on Temporal Distribution of NO3-N Leaching Losses to Subsurface Drainage Water. *Water Air Soil Pollut* **181**, 35–50 (2007).
16. Bakhsh, A., Kanwar, R. S. & Baker, J. L. N-Application Methods and Precipitation Pattern Effects on Subsurface Drainage Nitrate Losses and Crop Yields. *Water Air Soil Pollut* **212**, 65–76 (2010).
17. Helmers, M. J., Zhou, X., Baker, J. L., Melvin, S. W. & Lemke, D. W. Nitrogen loss on tile-drained Mollisols as affected by nitrogen application rate under continuous corn and corn-soybean rotation systems. *Can. J. Soil. Sci.* **92**, 493–499 (2012).
18. Jaynes, D. B. Nitrate loss in subsurface drainage and corn yield as affected by timing of sidedress nitrogen. *Agricultural Water Management* **130**, 52–60 (2013).
19. Jaynes, D. b., Colvin, T. s., Karlen, D. l., Cambardella, C. a. & Meek, D. w. Nitrate Loss in Subsurface Drainage as Affected by Nitrogen Fertilizer Rate. *Journal of Environmental Quality* **30**, 1305–1314 (2001).
20. Kucharik, C. J. & Brye, K. R. Integrated BIosphere Simulator (IBIS) Yield and Nitrate Loss Predictions for Wisconsin Maize Receiving Varied Amounts of Nitrogen Fertilizer. *Journal of Environmental Quality* **32**, 247–268 (2003).
21. Prunty, L. & Greenland, R. Nitrate leaching using two potato-corn N-fertilizer plans on sandy soil. *Agriculture, Ecosystems & Environment* **65**, 1–13 (1997).
22. Randall, G. W., Vetsch, J. A. & Huffman, J. R. Nitrate Losses in Subsurface Drainage from a Corn–Soybean Rotation as Affected by Time of Nitrogen Application and Use of Nitrapyrin. *Journal of Environmental Quality* **32**, 1764–1772 (2003).
23. Randall, G. W. & Vetsch, J. A. Nitrate Losses in Subsurface Drainage from a Corn–Soybean Rotation as Affected by Fall and Spring Application of Nitrogen and Nitrapyrin. *Journal of Environmental Quality* **34**, 590–597 (2005).

24. Sexton, B. T., Moncrief, J. F., Rosen, C. J., Gupta, S. C. & Cheng, H. H. Optimizing Nitrogen and Irrigation Inputs for Corn Based on Nitrate Leaching and Yield on a Coarse-Textured Soil. *Journal of Environmental Quality* **25**, 982–992 (1996).
25. Walters, D. T. & Malzer, G. L. Nitrogen Management and Nitrification Inhibitor Effects on Nitrogen-15 Urea: II. Nitrogen Leaching and Balance. *Soil Science Society of America Journal* **54**, 122–130 (1990).
26. Kalita, P. K., Algoazany, A. S., Mitchell, J. K., Cooke, R. A. C. & Hirschi, M. C. Subsurface water quality from a flat tile-drained watershed in Illinois, USA. *Agriculture, Ecosystems & Environment* **115**, 183–193 (2006).
27. O'Brien, P. L. *et al.* Nitrate losses and nitrous oxide emissions under contrasting tillage and cover crop management. *Journal of Environmental Quality* **51**, 683–695 (2022).
28. Gentry, L. E. *et al.* Split fertilizer nitrogen application with a cereal rye cover crop reduces tile nitrate loads in a corn–soybean rotation. *Journal of Environmental Quality* **53**, 90–100 (2024).
