# Supplementary material for: Environmental and societal costs of maize production decrease by addressing the uncertainty in nitrogen rate recommendations
Source: Nat Commun. 2026 Feb 5;17:2375. doi: 10.1038/s41467-026-68988-y (PMC12982502; doi:10.1038/s41467-026-68988-y)
Supplement: Supplementary file 3 — Reporting Summary [file 41467_2026_68988_MOESM3_ESM.pdf]

nature portfolio

Corresponding author(s): Francisco Palmero

Last updated by author(s): Dec 19, 2025

Reporting Summary

Nature Portfolio wishes to improve the reproducibility of the work that we publish. This form provides structure for consistency and transparency in reporting. For further information on Nature Portfolio policies, see our Editorial Policies and the Editorial Policy Checklist.

Statistics

For all statistical analyses, confirm that the following items are present in the figure legend, table legend, main text, or Methods section.

n/a	Confirmed	
☐	☒	The exact sample size (*n*) for each experimental group/condition, given as a discrete number and unit of measurement
☒	☐	A statement on whether measurements were taken from distinct samples or whether the same sample was measured repeatedly
☐	☒	The statistical test(s) used AND whether they are one- or two-sided<br>*Only common tests should be described solely by name; describe more complex techniques in the Methods section.*
☒	☐	A description of all covariates tested
☒	☐	A description of any assumptions or corrections, such as tests of normality and adjustment for multiple comparisons
☐	☒	A full description of the statistical parameters including central tendency (e.g. means) or other basic estimates (e.g. regression coefficient) AND variation (e.g. standard deviation) or associated estimates of uncertainty (e.g. confidence intervals)
☐	☒	For null hypothesis testing, the test statistic (e.g. *F*, *t*, *r*) with confidence intervals, effect sizes, degrees of freedom and *P* value noted<br>*Give P values as exact values whenever suitable.*
☐	☒	For Bayesian analysis, information on the choice of priors and Markov chain Monte Carlo settings
☒	☐	For hierarchical and complex designs, identification of the appropriate level for tests and full reporting of outcomes
☒	☐	Estimates of effect sizes (e.g. Cohen's *d*, Pearson's *r*), indicating how they were calculated

Our web collection on statistics for biologists contains articles on many of the points above.

Software and code

Policy information about availability of computer code

Data collection	The data used in this study was published by Ransom et al. (2021) and are publicly available at https://doi.org/10.5061/dryad.66t1g1k2g. Kitchen et al. (2017; https://acsess.onlinelibrary.wiley.com/doi/full/10.2134/agronj2017.04.0207) and Ransom et al. (2021; https://acsess.onlinelibrary.wiley.com/doi/full/10.1002/agj2.20812) provided details about sample sizes, experimental design, sampling protocol, plant and crop measurements, etc.
Data analysis	The central code (to fit the Bayesian quadratic plateau model) to the research presented in this study is available with open access via GitHub at https://github.com/FranciscoPalmero/AONR_EONR_uncertainty.git or through Zenodo at https://zenodo.org/doi/10.5281/zenodo.17868489.

For manuscripts utilizing custom algorithms or software that are central to the research but not yet described in published literature, software must be made available to editors and reviewers. We strongly encourage code deposition in a community repository (e.g. GitHub). See the Nature Portfolio guidelines for submitting code & software for further information.

Data

Policy information about availability of data

All manuscripts must include a data availability statement. This statement should provide the following information, where applicable:

- Accession codes, unique identifiers, or web links for publicly available datasets
- A description of any restrictions on data availability
- For clinical datasets or third party data, please ensure that the statement adheres to our policy

All data used in this study are publicly available. The dataset of maize grain yield response to N used in this study are available in the Ransom et al. database under accession code https://doi.org/10.5061/dryad.66t1g1k2g. The dataset of maize grain and fertilizer prices to define probability distribution of the price ratio used in this study are available in the USDA-Economic Research Survey under the accession codes https://www.ers.usda.gov/data-products/fertilizer-use-and-price/documentation-and-data-sources, and https://www.ers.usda.gov/data-products/season-average-price-forecasts. The literature review process about N-N2O emissions and N-NO_3^- leaching in the US Corn Belt data generated in this study are provided in the Supplementary Information file (Table S3). The data to build the plots presented in Fig. 3A, Fig. S1, Fig. S7, Fig. S8, and Fig. S9 can be reproduced from raw data and code that have already been shared in public repositories. The plots shown in Fig. 1 and Fig. 5 are illustrations, no data was used to build those figures. Source data are provided with this paper in Palmero et al. under accession code https://doi.org/10.6084/m9.figshare.30524483.

Research involving human participants, their data, or biological material

Policy information about studies with human participants or human data. See also policy information about sex, gender (identity/presentation), and sexual orientation and race, ethnicity and racism.

Reporting on sex and gender	na
Reporting on race, ethnicity, or other socially relevant groupings	na
Population characteristics	na
Recruitment	na
Ethics oversight	na

Note that full information on the approval of the study protocol must also be provided in the manuscript.

Field-specific reporting

Please select the one below that is the best fit for your research. If you are not sure, read the appropriate sections before making your selection.

☒ Life sciences ☐ Behavioural & social sciences ☐ Ecological, evolutionary & environmental sciences

For a reference copy of the document with all sections, see nature.com/documents/nr-reporting-summary-flat.pdf

Life sciences study design

All studies must disclose on these points even when the disclosure is negative.

Sample size	In the original dataset published by Ransom et al. (2021), there was two nitrogen fertilization timing. To avoid any confounded effect with application time, we selected the observations in which the nitrogen fertilizer rate was applied all at planting. Therefore, a total of 32 observations (n=32) were implemented in each site-year combination to fit the maize grain yield response to nitrogen fertilization. This sample size along with the range of explored nitrogen rates (from 0 to 315 kg/ha) are suitable for obtaining good statistical estimations of optimum nitrogen rates. For more details about sample size refer to Kitchen et al. (2017; https://acsess.onlinelibrary.wiley.com/doi/full/10.2134/agronj2017.04.0207) and Ransom et al. (2021; https://acsess.onlinelibrary.wiley.com/doi/full/10.1002/agj2.20812).
Data exclusions	No data was excluded to perform the analysis in this study. All the 49 sites-year combinations and all the observations (in which the nitrogen fertilizer rate was applied all at planting) within each combination presented in the original dataset were considered in this research.
Replication	49 replications of the same experimental protocol were performed in different locations in three consecutive years. All attempts at replication were successful. For more details about replications refer to Kitchen et al. (2017; https://acsess.onlinelibrary.wiley.com/doi/full/10.2134/agronj2017.04.0207) and Ransom et al. (2021; https://acsess.onlinelibrary.wiley.com/doi/full/10.1002/agj2.20812).
Randomization	Blocking was implemented to control spatial variability within a field in a given year. Therefore, nitrogen treatments were randomly assigned to plots within blocks. More information about site selection criteria, site characterization, experimental design, N fertilization treatments and implementation, use of common equipment, sample schedule, sample labeling, soil and plant sampling procedures, sample processing, sample storage, and data management can be found in Kitchen et al. (2017; https://acsess.onlinelibrary.wiley.com/doi/full/10.2134/agronj2017.04.0207) and Ransom et al. (2021; https://acsess.onlinelibrary.wiley.com/doi/full/10.1002/agj2.20812).
Blinding	Blinding of investigators to group allocation was not implemented during data collection or analysis. This study relied on secondary analysis of an open, publicly available dataset of maize grain yield responses to nitrogen rate, where treatment identities and nitrogen rates are intrinsic components of the data and are required to fit and interpret response curves. Because the analyses focused on modeling continuous

nitrogen–yield relationships rather than comparing discrete treatment groups or subjective outcome assessments, blinding was not feasible and was not considered relevant to the objectives of the study.

Reporting for specific materials, systems and methods

We require information from authors about some types of materials, experimental systems and methods used in many studies. Here, indicate whether each material, system or method listed is relevant to your study. If you are not sure if a list item applies to your research, read the appropriate section before selecting a response.

Materials & experimental systems

n/a	Involved in the study	
☒	☐	Antibodies
☒	☐	Eukaryotic cell lines
☒	☐	Palaeontology and archaeology
☒	☐	Animals and other organisms
☒	☐	Clinical data
☒	☐	Dual use research of concern
☐	☒	Plants

Methods

n/a	Involved in the study	
☒	☐	ChIP-seq
☒	☐	Flow cytometry
☒	☐	MRI-based neuroimaging

Dual use research of concern

Policy information about dual use research of concern

Hazards

Could the accidental, deliberate or reckless misuse of agents or technologies generated in the work, or the application of information presented in the manuscript, pose a threat to:

No	Yes	
☒	☐	Public health
☒	☐	National security
☒	☐	Crops and/or livestock
☒	☐	Ecosystems
☒	☐	Any other significant area

Experiments of concern

Does the work involve any of these experiments of concern:

No	Yes	
☒	☐	Demonstrate how to render a vaccine ineffective
☒	☐	Confer resistance to therapeutically useful antibiotics or antiviral agents
☒	☐	Enhance the virulence of a pathogen or render a nonpathogen virulent
☒	☐	Increase transmissibility of a pathogen
☒	☐	Alter the host range of a pathogen
☒	☐	Enable evasion of diagnostic/detection modalities
☒	☐	Enable the weaponization of a biological agent or toxin
☒	☐	Any other potentially harmful combination of experiments and agents

Plants

Seed stocks	na
Novel plant genotypes	na
Authentication	na
